# Tear Film Osmolarity in Horses With Bacterial Conjunctivitis

**DOI:** 10.1002/vms3.70677

**Published:** 2025-11-17

**Authors:** Ahad Saberinia, Saeed Ozmaei, Amin Anoushepour

**Affiliations:** ^1^ Department of Clinical Sciences, Science and Research Branch Islamic Azad University Tehran Iran; ^2^ Department of Veterinary Clinical Sciences, Ka.C. Islamic Azad University Karaj Iran

**Keywords:** bacterial conjunctivitis, equine ophthalmology, osmolarity, tear film

## Abstract

**Background:**

Conjunctivitis is an inflammation of the conjunctiva caused by infectious or non‐infectious factors. Infectious conjunctivitis comes in two forms: viral and bacterial. Bacterial conjunctivitis is commonly caused by organisms such as *Staphylococcus* and *Streptococcus*, with the severity of the disease influenced by the specific bacterial species involved. Because both viral and bacterial conjunctivitis are highly contagious, preventive measures are essential to reduce transmission—especially to the unaffected eye.

**Objective:**

To evaluate the tear film osmolality in horses with bacterial conjunctivitis.

**Methods:**

A total of 40 healthy horses and those with conjunctivitis were included in the study. Tear samples were collected from both eyes with microcapillary tubes three times at 5‐min intervals. The tear samples for each horse were pooled, and the osmolality and electrolyte concentrations were measured. The mean (SD) was calculated for each variable to establish preliminary guidelines for tear film osmolality and electrolyte composition in healthy horses.

**Results:**

The mean (SD) tear film osmolality was 283.51 (9.33) mmol/kg, and the mean (SD) sodium, potassium, magnesium and calcium concentrations were 134.75 (10), 16.3 (5.77), 3.48 (1.97) and 1.06 (0.42) mmol/L, respectively. The sodium concentration in the tear film was similar to that in serum, whereas the potassium concentration in the tear film was approximately 4.75 times that of serum.

**Conclusions:**

Measuring tear‐film osmolality offers a non‐invasive, easy and practical approach to gaining valuable insights into baseline conditions and potential changes in ocular diseases. However, its true clinical utility requires integration with other ocular parameters and further controlled studies in horses suffering from conjunctivitis. The development of such tools could lead to more accurate diagnoses and more comprehensive treatment strategies.

## Graphical abstract

This study evaluated tear film osmolarity in horses with bacterial conjunctivitis. Although osmolarity values were higher in affected horses compared to healthy ones, the difference was not statistically significant. These findings highlight the need for further research before tear osmolarity can be considered a reliable diagnostic marker in equine ophthalmology.

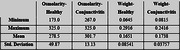

## 1 Introduction

Tear osmolarity is a critical diagnostic parameter for ocular surface diseases. While its role is well understood in dry eye syndrome and corneal disorders, its relevance in bacterial conjunctivitis—particularly in horses—remains unclear. This study investigates whether tear osmolarity changes significantly in equine bacterial conjunctivitis and assesses its potential as a diagnostic marker.

Tear osmolarity depends on both tear secretion and evaporation rates. That measuring osmolarity in tears can aid diagnosis and management of various ocular conditions, including dry eye, by tracking tear stability, secretion and disease progression (Bron and Willshire 2021; Gilbard and Farris 1979; Potvin et al. 2015).

Specifically in horses, establishing baseline osmolarity is essential: Best et al. (2015) reported an average of 283.51 ± 9.33 mmol/kg in healthy equines (Best et al. 2015).

Conjunctivitis is an ocular inflammatory reaction that comes with swelling, redness, itching and eye discharge. It can occur with many conditions, such as infections, allergies or contact with an irritant (Brooks 2010).

Bacteria can be cultured from the conjunctival sac in 46%–90% of healthy animals. Aerobic gram‐positive bacteria are the most cultured (Maggs et al. 2017). Bacterial conjunctivitis in horses is commonly caused by *Moraxella equi*, *Streptococcus equi* ssp. equi, *Rhodococcus equi* sp., *Actinobacillus* sp. and *Leptospira* sp. (Brooks 2010).

Inflammatory processes alter tear composition by increasing solute concentration—due to reduced tear flow and changes in protein and electrolyte balance—resulting in elevated osmolarity (Bron et al. 2017; Tashbayev et al. 2020).

Accordingly, the purpose of this study is to determine whether there is a measurable correlation between tear osmolarity and bacterial conjunctivitis in horses, to identify this inflammation early and potentially incorporate osmolarity testing into clinical practice.

## 2 Materials and Methods

### 2.1 Animal Selection and Inclusion Criteria

A total of 40 horses (11 months to 15 years old) were selected. Out of which 20 horses had clinical signs of conjunctivitis (ocular discharge, hyperaemia, chemosis and photophobia) and positive bacterial cultures. Other 20 clinically healthy horses served as controls.

### 2.2 Sample Collection and Bacterial Identification

**FIGURE 1:**
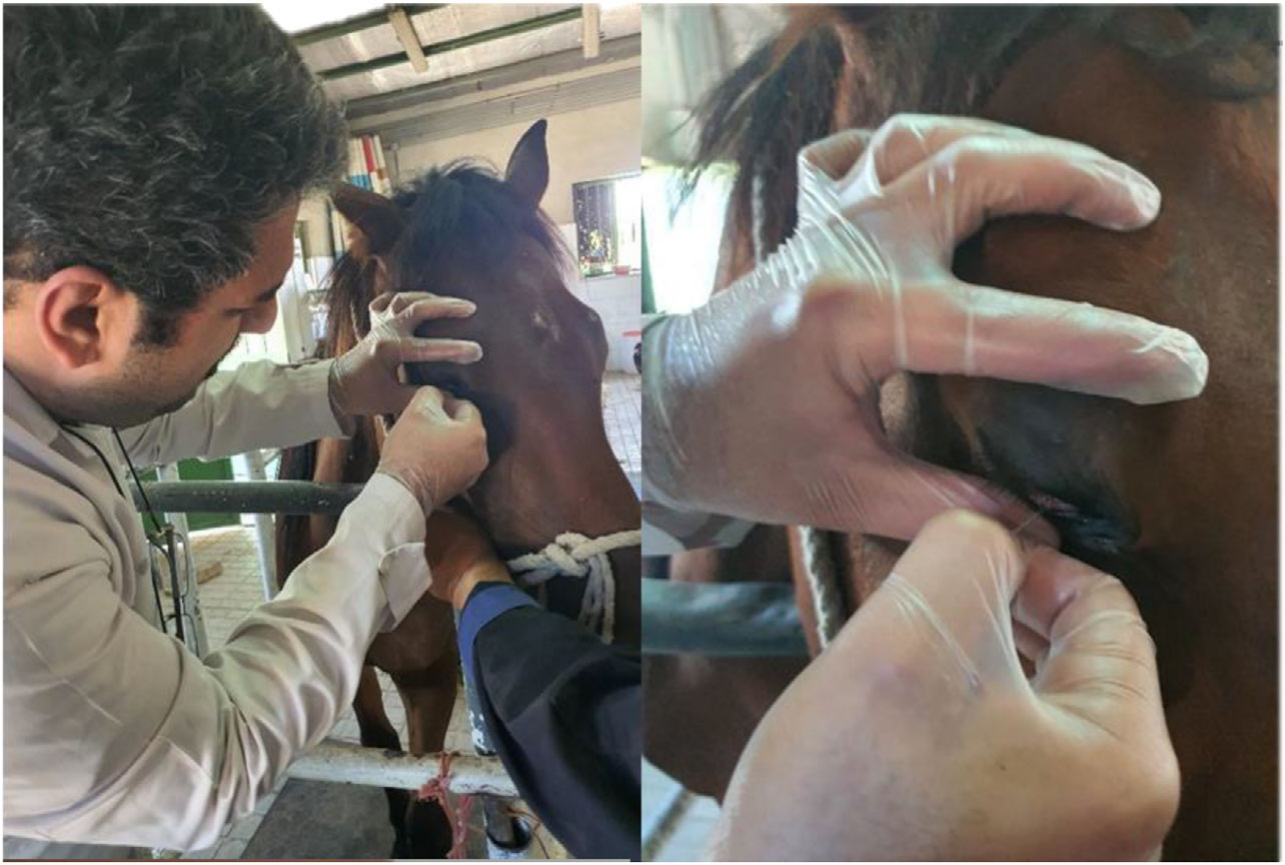
Tear sample collection from equine eye using sterile microcapillary tube.

Tear samples were collected using sterile microcapillary tubes, as illustrated in Figure 1. Bacterial cultures were performed using Blood Agar, MacConkey Agar, SIM, TSI and Simon Citrate media.

Because drugs can influence our results, we did not use any sedation or other drugs in this study. After collecting samples, we used the fluorescence paper method to examine the eyes to make sure our study did not cause any eye damage, such as corneal ulcers, et cetera.

### 2.3 Tear Osmolarity Measurement

Osmolarity was measured using an OSMOMAT 030 osmometer with a minimum tear volume of 50 µL per sample, collected at 5‐min intervals.

To get this volume of the sample using a haematocrit tube, we put the tube at the medial canthus of each horse eye three times, 5 min apart. In order to achieve comprehensive representation, the principles of capillarity law allow us to carefully draw liquids into a special tube with the help of natural forces. The amount of haematocrit tube is 70 µL, but in order to achieve comprehensive representation, we take 210 µL, pour them into a microtube and bring them to the lab near ice.

The data obtained from the samples were analysed statistically using SPSS software, descriptive statistics and ANOVA tests.

## 3 Result

In order to find 20 horses with bacterial conjunctivitis, the number of cultured samples in culture mediums reached 37 samples, In general, in this study, we were looking for the conjunctivitis that we found to have a bacterial cause, but for better confirmation, we also determined the type of bacteria using differential culture media, out of these 20 samples, 6 samples were of the species *Actinobacillus*, 5 samples of *Leptospira*, 6 samples of *Streptococcus equi*, 2 samples of *Rhodococcus equi* and 1 sample of *Moraxella* were found.

Overall, horses with bacterial conjunctivitis examined in this experiment were, on average, about 8 years old (range, 4–15 years). The amount of tears obtained from these horses was, on average, about 173 µL (range of values from 80 to 241 µL). The osmolarity obtained from these tear samples was, on average, about 301 mOsm (range of values from 267 to 325 mOsm).

**FIGURE 2:**
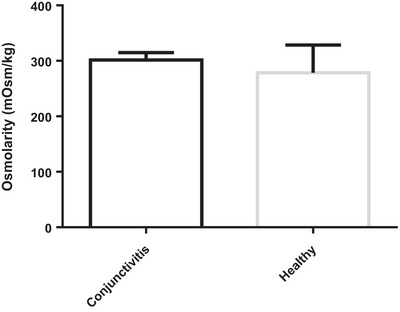
Comparison chart of osmolarity based on group mean.

In contrast, control and healthy horses, which were considered a comparison group, were on average about 6 years old (age range from 11 months to 11 years). The amount of tears obtained from these horses was, on average, about 165 ± 7 µL (range of values from 64 to 291 µL). This group of horses had an average osmolarity of roughly 278.5 ± 23.4 mOsm (with a range of values from 173 to 325 mOsm). A summary of the key finding is provided in Table 1 and Figure 2.

**TABLE 1:** Descriptive review of results.

	Osmolarity‐healthy	Osmolarity‐conjunctivitis	Weight‐healthy	Weight‐conjunctivitis
Minimum	173.0	267.0	0.0645	0.0815
Maximum	325.0	325.0	0.2916	0.2416
Mean	278.5	301.7	0.1651	0.1738
Std. Deviation	49.87	13.13	0.08541	0.03757

The osmolarity of healthy horses and horses with conjunctivitis does not differ statistically significantly, according to the statistical results (*p* > 0.05).

## 4 Discussion

In this study, the mean tear film osmolarity in horses diagnosed with bacterial conjunctivitis was reported as 278.5 ± 23.4 mOsm/kg. This value is comparable to reference ranges previously reported for healthy horses in some studies, although the observed variability may be associated with inflammatory changes and increased tear evaporation under pathological conditions. For a more thorough analysis, the results of this study were compared with several similar investigations:

In a study conducted by Best et al. 2015, aimed at evaluating the osmolarity and electrolyte composition of the tear film in 15 healthy adult horses, the mean tear film osmolarity was 283.51 ± 9.33 mOsm/kg. This value falls within the range reported by previous studies for healthy horses. Tear osmolarity in both healthy horses and those with mild ocular disorders ranged from 265 to 290 mOsm/kg. These findings provided a preliminary reference framework for comparing tear samples from horses affected by corneal diseases. Measuring tear film osmolarity in these horses was simple and non‐invasive. Clinically, this data could be useful for diagnosing and monitoring ocular surface diseases associated with elevated osmolarity in horses.

In a study by Almutleb et al. (2025), the tear film of Arabian horses with clinically healthy eyes was compared to that of healthy human eyes. A total of 94 Arabian horses and 94 human participants were included. Tear film parameters for the right eye of each participant were evaluated using the EASYTEAR View+ device, with 5‐min intervals between each test. Each test was repeated three times by the same examiner. Statistical analysis revealed a significant difference in Non‐Invasive Tear Breakup Time (NIBUT) (*p*  =  0.001) and Lipid Layer Pattern (*p*  =  0.010) between horses and humans. Based on these results, it can be concluded that horses have a longer tear film breakup time and a thicker lipid layer compared to healthy human eyes, while their tear meniscus height was similar.

Davis and Townsend (2011) conducted a study comparing tear film osmolarity in healthy cats and cats with conjunctivitis. A total of 93 cats were examined—37 were healthy and 39 had conjunctivitis. The average age was 2.34 years. The study found no statistically significant difference in mean tear osmolarity between healthy cats (328.5 ± 17.94 mOsm/kg) and those with conjunctivitis (325 ± 24.84 mOsm/kg), which aligns with the findings of the current study.

In a study by Lamkin et al. (2020), aimed at comparing basal and reflex tear osmolarity in healthy dogs and evaluating correlations among osmolarity, tear production and tear fern pattern, results indicated no difference between basal and reflex tear osmolarity. A weak positive correlation was observed between tear production and reflex tear osmolarity. However, no correlation was found between osmolarity measured using a handheld osmometer and the tear fern pattern—a finding consistent with our present results.

These findings suggest that bacterial conjunctivitis in its early or moderate stages does not lead to significant changes in tear osmolarity. However, a study conducted by Sebbag et al. (2016) reported a significant increase in osmolarity in chronic or severe inflammatory ocular conditions, possibly due to species‐specific immune responses and variations in tear evaporation rates.

The results of this study, along with similar research, indicate that osmolarity alone may not serve as a definitive marker for diagnosing the severity of bacterial conjunctivitis in horses. However, it may be a valuable tool when used alongside other clinical tests and imaging techniques for monitoring disease progression.

Overall, the findings of this study are relatively consistent with previous reports and normal reference values. This suggests that in mild to moderate bacterial conjunctivitis, osmolarity changes may not be significant. However, in more severe or chronic conditions, greater changes in osmolarity should be expected. Given that osmolarity measurement is a quick and non‐invasive procedure, it holds potential value in the diagnosis and follow‐up of equine ocular diseases.

One of the limitations of this study was the short‐term use of infected horses and the single‐time examination, which may not fully reflect the real impact of conjunctivitis on tear osmolarity in horses. Broader and longer‐term studies are necessary to confirm this hypothesis. The use of only one horse breed and the small sample size also posed limitations. As a result, this study could not determine the specific effects of individual bacterial species on tear osmolarity. Therefore, we have reported the overall effect of bacterial conjunctivitis on tear osmolarity. To evaluate the impact of each bacterial species, larger sample sizes are needed to allow comparisons between at least two bacterial pathogens. Further long‐term research involving horses affected by conjunctivitis and different bacterial strains is essential.

## 5 Conclusion

The osmolarity values in horses with bacterial conjunctivitis remained within the physiological range. This may suggest that inflammatory responses in the early or mild stages of the disease do not significantly affect tear film osmolarity. However, further studies that consider disease severity, duration and comparisons with non‐infectious ocular conditions in horses are needed to confirm this observation.

## Author Contributions

**Ahad Saberinia**: sample collection, conducted the experiments, data analysis, original draft of the manuscript. **Saeed Ozmaei**: conceived and designed, sample collection, data analysis, reviewed and edited the manuscript. **Amin Anoushepour**: conceived and designed, sample collection, reviewed and edited the manuscript. All authors contributed to the design of the study, as well as data collection and analysis and the writing of the manuscript. All authors read and approved the final manuscript.

## Funding

The authors have nothing to report.

## Ethics Statement

The authors confirm that the ethical policies of the journal have been adhered to and that appropriate ethical review committee approval has been obtained (Approval Number: **IR.IAU.SRB.REC.1401.347**). The study followed the US National Research Council's guidelines for the Care and Use of Laboratory Animals.

## Conflicts of Interest

The authors declare no conflicts of interest.

## Peer Review

The peer review history for this article is available at https://doi.org/10.1002/vms3.70677.

## Data Availability

The data that support the findings of this study are available on request from the corresponding author.
